# Supplementary figures and images for: Transcriptional Analysis Reveals the Differences in Response of Floral Buds to Boron Deficiency Between Two Contrasting Brassica napus Varieties
Source: Plants (Basel). 2025 Mar 10;14(6):859. doi: 10.3390/plants14060859 (PMC11944869; doi:10.3390/plants14060859)

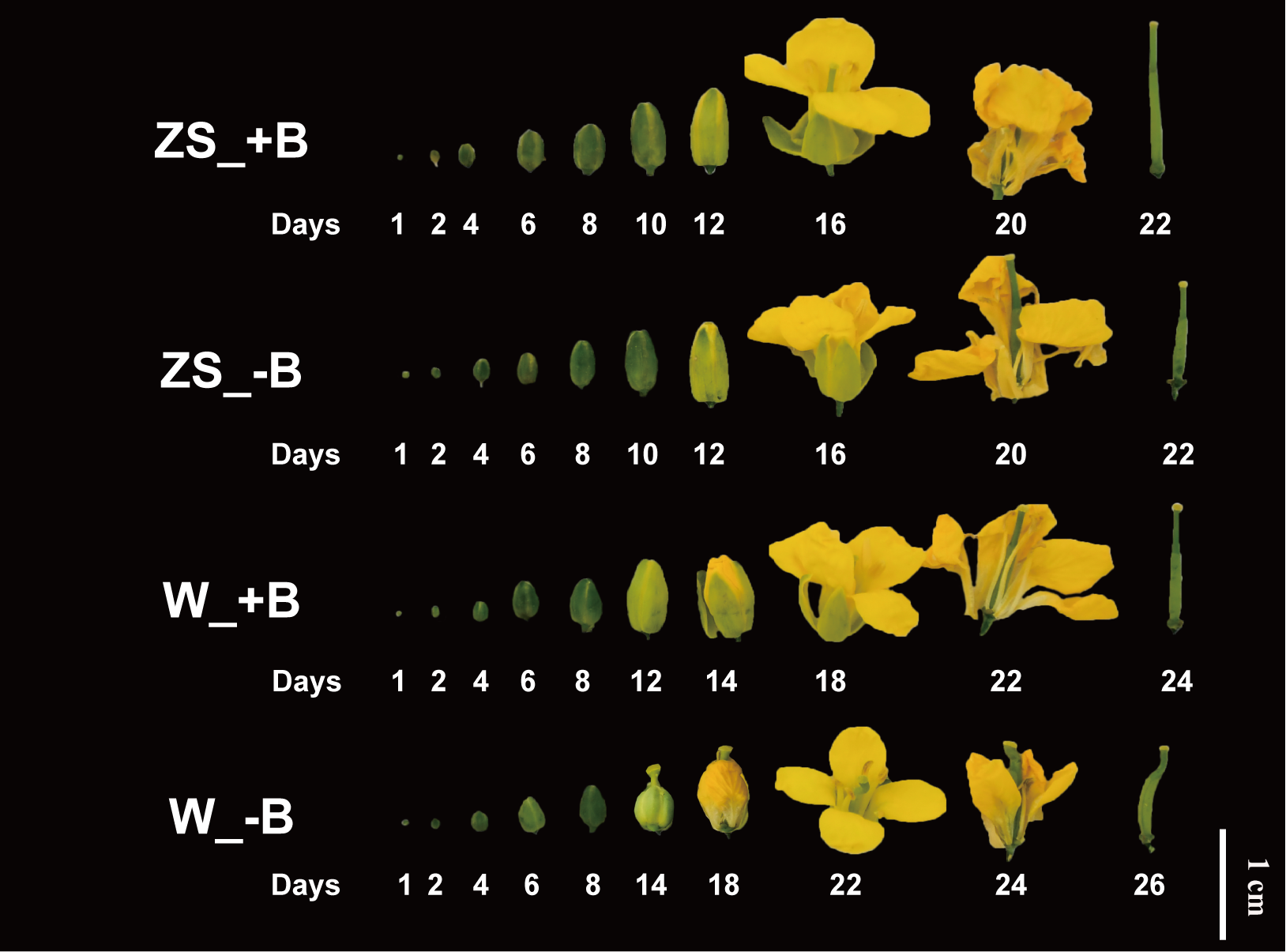

Supplement: Supplementary file 1 [file plants-14-00859-s001.zip › Figure S1.tif]
